# Acute poisoning with acetamiprid: a case report

**DOI:** 10.1186/s13256-021-02919-x

**Published:** 2021-07-30

**Authors:** Selladurai Pirasath, Rajaram Senthuran, Chandrakulasegeran Athirayan, Mathyasekeran Gevakaran, Mahesan Guruparan, Ariaranee Gnanathasan

**Affiliations:** 1 District General Hospital, Kilinochchi, Sri Lanka; 2 Teaching Hospital, Jaffna, Sri Lanka; 3 Faculty of Medicine, University of Colombo and National Hospital of Colombo, Colombo, Sri Lanka; 4 Ilavalai North, Ilavalai, Jaffna, Sri Lanka

**Keywords:** Acetamiprid, Lactic acidosis, Myocardial ischemia, Hypotension, Hypokalemia

## Abstract

**Background:**

Acetamiprid is a potent new first-generation neonicotinoid insecticide in agricultural practices. It is well described that it has low toxicity among animals and is lethal if consumed in large amounts. However, toxicity in humans is rarely reported in literature. Here, we describe acetamiprid toxicity complicated with severe lactic acidosis, myocardial ischemia, refractory hypotension, and severe hypokalemia in a middle-aged man who presented with deliberate self-harming with acetamiprid poisoning in Sri Lanka.

**Case presentation:**

We describe a case of acute poisoning with an acetamiprid in a middle-aged Sri Lankan Tamil farmer for suicidal purposes following family conflicts with his wife. He presented with severe nausea, vomiting, and altered level of consciousness. He had electrocardiogram changes, hypoxia, and lactic acidosis. With intensive care management including ventilatory support, inotropic therapy along with intraarterial blood pressure monitoring, correction of acidosis, and administration of electrolytes, he made good clinical recovery. He was discharged without any further complications 6 days after ingestion of acetamiprid.

**Conclusions:**

This case illustrates a rare, acute poisoning with acetamiprid in human, as well as its clinical manifestations and successful management with supportive therapy. This will be helpful for clinicians to identify clinical manifestations and to guide management of acute poisoning with acetamiprid in the future.

## Background

Acetamiprid belongs to a new systemic neonicotinoid insecticide that is effectively used for crop protection and flea control in agricultural works [1]. It has low toxicity in mammals, but ingestion of large amounts can cause severe toxicity. It is described in a case report that a buffalo exhibited severe gastrointestinal symptoms and respiratory distress following accidental ingestion of acetamiprid in India [2]. Furthermore, only two cases of ingestion of acetamiprid poisoning in human have been reported in literature [3, 4]. Here, we describe a third case of acetamiprid toxicity complicated with severe lactic acidosis, myocardial ischemia, refractory hypotension, and severe hypokalemia in a middle-aged man following deliberate self-harming with acetamiprid in Sri Lanka.

## Case presentation

**Fig. 1:**
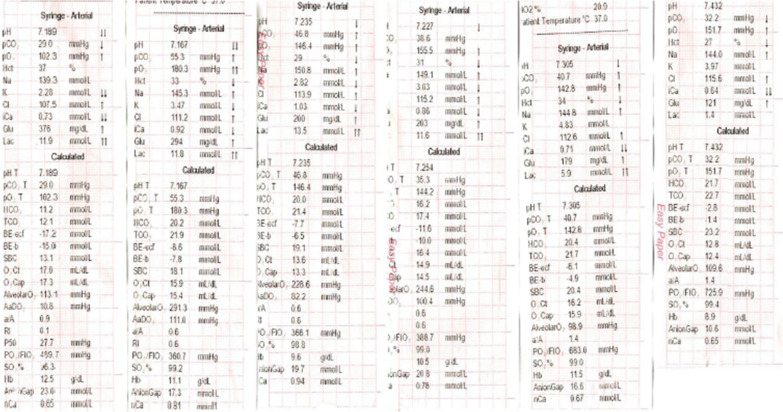
Serial arterial blood gas reports of patient with acetamiprid poisoning

**Fig. 2:**
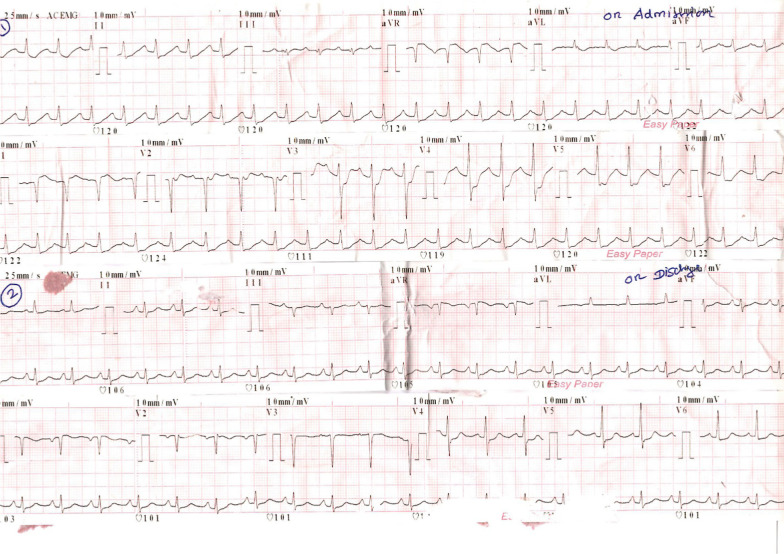
The 12 lead electrocardiography (ECG) showed ST depression in I, II, V_3_–V_6_ on admission and resolution of ECG changes on discharge in a patient with acetamiprid poisoning.

**Fig. 3:**
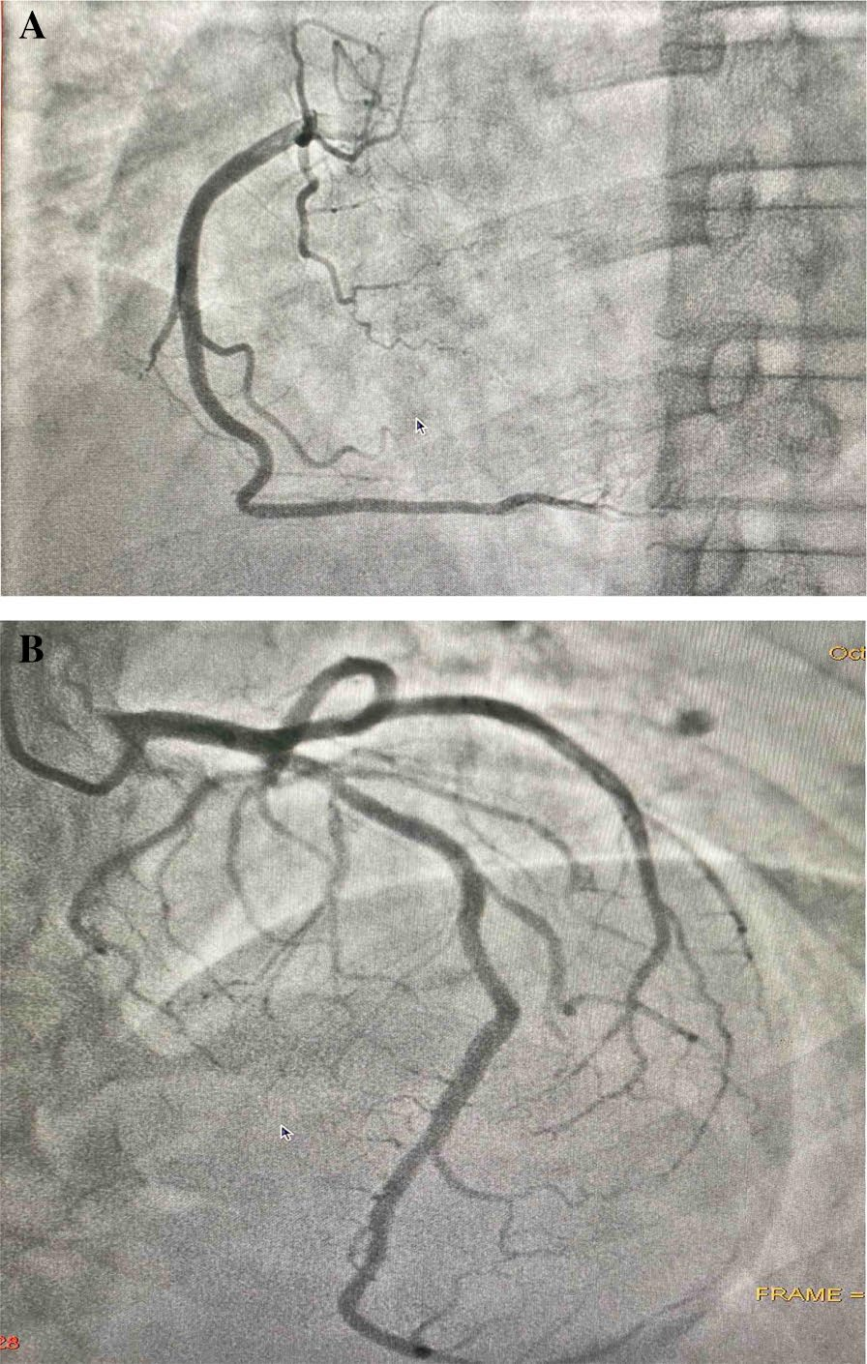
**A**, **B** Coronary angiography showing normal right (**A**) and left coronary arteries (**B**) in a patient with acetamiprid poisoning

A 58-year-old healthy Tamil farmer was admitted to the emergency unit of a district general hospital in Northern Sri Lanka with history of nausea, vomiting, and altered level of consciousness 1 hour after acetamiprid poisoning. He had ingested 150 g of acetamiprid dissolved in water. On examination, he was conscious with Glasgow Coma Scale score of 14/15 (GCS 14/15), and both pupils were equally reactive to light and 3 mm in size. His blood pressure was 112/65 mmHg, pulse 86 beats per minute, respiratory rate 12 breaths per minute, and core body temperature 36.8°C. The rest of the systemic examinations were unremarkable. Gastric lavage was performed, and a single dose of activated charcoal was administrated. One hour later, he developed reduced level of consciousness with GCS of 8/15. His oxygen saturation dropped to 75%, and blood pressure was 60/40 mmHg. His arterial blood gas was tested (Fig. 1), showing severe lactic acidosis (pH 7.18, PaCO_2_ 29 mmHg, PaO_2_ 102.3 mmHg, HCO_3_^−^ 11.2 mEq/L, blood lactate 11.9 mmol/L). To correct acidosis, 100 ml of 8.4% intravenous sodium bicarbonate was given. He was electively intubated. Subsequently, he was given fluid boluses of 30 ml/kg, such as 0.9% saline 2 L, 2% albumin saline 500 ml, and 5% human albumin 250 ml, which was guided according to dynamic parameters of fluid responsiveness such as inferior vena cava distensibility and right ventricular collapse in imaging studies. However, his blood pressure was suboptimal. Therefore, noradrenaline infusion at rate of 0.1 μg/kg/minute was initiated. His 12-lead electrocardiography (ECG) showed ST depression in leads I, II, aVF, and V_3_–V_6_ (Fig. 2). His high-sensitivity troponin I was 571 ng/L. Two-dimensional echocardiography (2D Echo) was done, and it showed mild left ventricular dysfunction with ejection fraction of 45%. Blood results are presented in Table 1. He had persistent hypotension of 70/40 mmHg and severe lactic acidosis despite therapy (Fig. 1). Subsequently, ultrasound-guided central venous line and intraarterial line were inserted. He was given dobutamine at rate of 10 μg/kg/minute followed by adrenaline infusion of 0.1 μg/kg/minute and vasopressin 1 U/kg/minute. Despite four inotropes, his blood pressure was suboptimal, and the infusion of noradrenaline, dobutamine, adrenaline, and vasopressin was increased to maximum doses of 0.7 μg/kg/minute, 20 μg/kg/minute, 1 μg/kg/minute, and 3 U/kg/minute, respectively. His blood pressure was maintained with infusion of four inotropes for 24 hours. Furthermore, 100 ml, 100 ml, and 50 ml of 8.4% intravenous sodium bicarbonate was repeated to correct acidosis according to calculated deficit and 100 ml/kg of 0.9% normal saline as maintenance therapy for the next 36 hours. He had persistent hypokalemia that required intravenous KCl of 60 mmol over 4 hours, 60 mmol for 3 hours, and 20 mmol for 1 hour. At end of 36 hours, his blood pressure and lactate level were brought to normal level. He was ventilated for 72 hours in the intensive care unit with further supportive therapy. He gradually improved and was extubated on day 3 of his illness. His repeat ECG and 2D Echo showed no abnormalities (Fig. 2). His coronary angiography showed normal coronary epicardial arteries (Fig. 3). He was discharged from hospital on day 6 of hospital admission.

**Table 1:** Biochemical profile of patient with clinical progression of disease

Biochemical investigation				Date		
	Day 1	Day 2	Day 3	Day 4	Day 5	Day 6
Full blood count						
White cell count (4000–11,000/mm^3^)	9540	10,400	10,600	11,320	10,324	10,321
Neutrophils (50–70%)	67	80	87	76	69	75
Lymphocytes (20–40%)	32	14	7	23	21	23
Hemoglobin (12–16 g/dL)	10.5	10.4	10.6	10.5	11.0	10.7
Red cell count (400,000–550,000 mm^3^)	376,000	352,000	394,000	375,000	321,000	311,000
Platelets (150,000–450,000 mm^3^)	303,000	246,000	342,000	327,000	298,000	276,000
Inflammatory markers						
ESR (first hour)	–	–	45	–	.	35
CRP (0–1.0 mg/dL)	–	3.1	2.1	1.7	1.1	1.1
Renal function tests						
Blood urea (18–55 mg/dL)	30	23	35	23	35	40
Serum creatinine (0.7–1.5 mg/dL)	1.17	1.51	1.83	1.64	1.1	1.1
Serum electrolytes						
Serum sodium (135–145 mmol/L)	145.2	138.1	139.1	134.9	135.6	134.8
Serum potassium (3.5–5.0 mmol/L)	2.39	2.4	3.1	4.2	5.2	4.5
Serum calcium (8.6–10.2 mg/dL)	–	8.2	8.2	8.7	8.5	8.4
Serum phosphorus (2.6–4.5 mg/dL)	–	3.5	3.1	2.7	2.8	2.3
Liver profile						
Serum aspartate aminotransferase (AST) (0–45 U/L)	19	61	188	123	72	47
Serum alanine aminotransferase (ALT) (0–35 U/L)	15	62	68	34	45	39
Serum bilirubin (0–2.0 mg/dL)	5.6	6.14.	6.13	4.1	3.1	2.1
Serum protein (6.4–8.3 g/dL)	5.59	5.12	5.0	5.8	5.9	5.8
Clotting profile						
Prothrombin time/international normalized ratio (PT/INR) (< 1.4)	–	1.4	–	1.1	–	1.0
Activated partial thromboplastin time (APTT) (< 35 s)	–	33	–	34	–	35
High-sensitivity troponin I (< 19 ng/L)	571	–	240.8	133.6	–	18

AST: Asparate transaminase, ALT: Alanine transaminase, PT/INR: Prothrombin time/International Normalized Ratio, APTT: Activated partial thromboplastin time

## Discussion and conclusions

Acetamiprid belongs to a new generation of neonicotinoid insecticides that act as selective agonists at nicotinic acetylcholine receptors [5]. Acetamiprid, clothianidin, dinotefuran, imidacloprid, nitenpyram, thiacloprid, and thiamethoxam are common insecticides belonging to this group. Several cases of imidacloprid toxicity were reported in Sri Lanka [6]. However, poisoning with acetamiprid is rare in humans and is not reported in Sri Lanka because neonicotinoids were introduced in market just a few decades ago [7]. Acetamiprid and imidacloprid account for 25% of global marketing insecticide, but are rarely reported in literature on humans [3, 4, 8]. Although the toxicity for insects is higher than for humans, in humans it causes neuromuscular paralysis and even death [9]. The toxicity can occur through inhalation, ingestion, and dermal contact.

The clinical presentation is similar to that of acute nicotine poisoning, but without corrosive injuries of gastrointestinal tract [5]. The cardiac, respiratory, and neurological symptoms of severe neonicotinoid intoxication are seen among animals [10]. In humans, the toxicity causes nausea, vomiting, respiratory failure, tachycardia, hypotension, muscle weakness, and convulsions [3]. Here, we described a case of acute acetamiprid poisoning in a middle-aged farmer who presented with nausea, vomiting, and altered level of consciousness 1 hour after ingestion.

Acetamiprid reaches high concentrations in liver, kidney, and adrenal gland after extensive absorption from gut in rats [11]. This may be the reason for the increased level of serum bilirubin in the presented case. Electrocardiographic changes noted in the literature include sinus tachycardia, cardiac arrhythmias, and ischemic changes [12]. Our patient developed sinus tachycardia and ischemic changes in ECG, as shown in Fig. 2.

The severity of toxicity has no positive correlation with plasma concentration of neonicotinoid concentration. Thus, hemoperfusion has no role in eliminating the toxicity. Supportive therapy and decontamination are recommended among all neonicotinoid-poisoned patients. Gastric lavage was performed, and activated charcoal was given to our patient. He developed severe hypotension, lactic acidosis, hypoxia, and reduced level of consciousness, which required ventilatory support, inotropes, and further supportive therapy. Thirty-six hours later, his symptoms with regard to the effects of acetamiprid were improved, and he was continued on inotropes and ventilator support for 3 days and discharged from hospital on day 6 without any complications.

In the case of acute toxicity, respiratory failure and reduced level of consciousness are the most serious but uncommon complications. The clinical consequences of acetamiprid poisoning are not very well described. Therefore, such information is valuable for clinicians, regulatory authorities, and the public at large. Furthermore, clinical outcomes depend on early recognition and aggressive supportive management since there is no antidote available.

## Data Availability

All the data generated or analyzed during this study are included in this article and its supplementary information files.

## Abbreviations

GCS: Glasgow Coma Scale
ECG: Electrocardiography
2D Echo: Two-dimensional echocardiography
ESR: Erythrocyte sedimentation rate
CRP: C-reactive protein
